# Revealing the Demographic History of the European Nightjar (*Caprimulgus europaeus*)

**DOI:** 10.1002/ece3.70460

**Published:** 2024-10-26

**Authors:** George Day, Graeme Fox, Helen Hipperson, Kathryn H. Maher, Rachel Tucker, Gavin J. Horsburgh, Dean Waters, Kate L. Durant, Terry Burke, Jon Slate, Kathryn E. Arnold

**Affiliations:** ^1^ Department of Environment and Geography University of York York UK; ^2^ NERC Environmental "Omics Facility ‐ Visitor Facility School of Biosciences Sheffield UK; ^3^ British Antarctic Survey Cambridge UK; ^4^ University of Nottingham Nottingham UK

**Keywords:** *Caprimulgus europaeus*, conservation genomics, demography, PSMC, refugia

## Abstract

A species' demographic history gives important context to contemporary population genetics and a possible insight into past responses to climate change; with an individual's genome providing a window into the evolutionary history of contemporary populations. Pairwise sequentially Markovian coalescent (PSMC) analysis uses information from a single genome to derive fluctuations in effective population size change over the last ~5 million years. Here, we apply PSMC analysis to two European nightjar (*Caprimulgus europaeus*) genomes, sampled in Northwest and Southern Europe, with the aim of revealing the demographic history of nightjar in Europe. We successfully reconstructed effective population size over the last 5 million years. Our analysis shows that in response to global climate change, the effective population size of nightjar broadly increased under stable warm periods and decreased during cooler spans and prolonged glacial periods. PSMC analysis on the pseudo‐diploid combination of the two genomes revealed fluctuations in gene flow between ancestral populations over time, with gene flow ceasing by the last‐glacial period. Our results are tentatively suggestive of divergence in the European nightjar population, with timings consistent with differentiation being driven by restriction to different refugia during periods of glaciation. Finally, our results suggest that migratory behaviour in nightjar likely evolved prior to the last‐glacial period, with long‐distance migration seemingly persisting throughout the Pleistocene. However, further genetic structure analysis of individuals from known breeding sites across the species' contemporary range is needed to understand the extent and origins of range‐wide differentiation in nightjar.

## Graphical abstract

We used pairwise sequentially Markovian coalescent analysis to reveal the ancient demographic history of two contemporary European nightjar (*Caprimulgus europaeus*) populations. We show that nightjar effective population size has fluctuated over the last 5 million years in relation to changes in global climate. We further show that restriction to glacial refugia likely led to divergence within the European population, with long‐distance migration seemingly persisting throughout the Pleistocene.

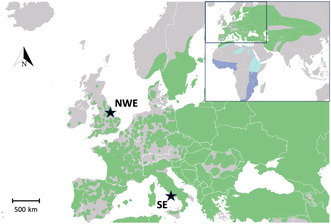

## 1 Introduction

Genomes provide a repository from which information on historic changes in genetic diversity, effective population size (*N*_e_), speciation, and population structuring can be inferred and used to track adaptations to environmental change (Mather, Traves, and Ho 2019; Patil and Vijay 2021). Specifically, sequence data from a single aligned genome can be used to track historic demographic patterns exhibited by a species or population (Li and Durbin 2011). Pairwise sequentially Markovian coalescent (PSMC) analysis is a powerful tool which infers ancestral changes in the effective population size (*N*_e_) from a single genome, from a single contemporary individual. The analysis applies hidden‐Markov modelling to the coalescence framework, treating a genome as multiple historic genealogies partitioned by recombination events (see Li and Durbin 2011; Mather, Traves, and Ho 2019 for detailed explanation of the method). PSMC analysis has been used to determine ancestral (up to ~5 Mya) population demographic trends from single genomes (e.g., Nadachowska‐Brzyska et al. 2015; Fuchs, Ericson, and Irestedt 2024). The analysis can be further applied to pseudo‐diploid genomes constructed from two individuals from different species or populations to investigate changes in gene flow and timing of divergence (Li and Durbin 2011; e.g., *Ficedula* flycatchers; Nadachowska‐Brzyska et al. 2016, *Catharus* thrushes; Termignoni‐Garcia et al. 2022). For example, PSMC applied to pseudo‐diploid genomes from three Golden eagle (*Aquila chrysaetos*) subspecies revealed the timing of divergence and gene flow change among subspecies over a time scale of ~11 million years from only three pseudo‐diploid genomes (Sato et al. 2020). When combined with geological and paleoclimate data, PSMC analysis can reflect a species' past ability to adapt to environmental change, and how different populations, or species, have been affected by ancient global climate trends (Nadachowska‐Brzyska et al. 2015; Mather, Traves, and Ho 2019). Understanding a species' response to past environmental change aids predictions to be made regarding vulnerability to contemporary and future climate change and how this may vary interspecifically under different life histories (Kozma et al. 2018, 2016; Chattopadhyay et al. 2019), or between populations at different locations across a species range (Sato et al. 2020).

Over the past ~5 million years the global climate has fluctuated dramatically, oscillating between periods of extensive glaciation and interglacial warming. Long glacial and short interglacial periods during the mid‐Pleistocene revolution (MPR; ~1 Mya–450 Kya) resulted in cooler interglacial temperatures than those presently recorded (Pisias and Moore 1981). However, throughout the Mid‐Brunhes Event (MBE; ~450–110 Kya), interglaciations were characterised by warmer temperatures, with comparatively less severe glacial periods compared with the mid‐Pleistocene (Candy et al. 2010; Barth et al. 2018). During the Last Glacial Period (LGP; ~110 Kya), the Fennoscandian ice sheet covered much of Western and Northwestern Europe, restricting temperate zones to contemporary Southern Eurasia (Denton and Hughes 1981). These significant shifts in global climate have been shown to correspond with fluctuations in historic population size in a number of species (Nadachowska‐Brzyska et al. 2015; Kozma et al. 2016, 2018). Over periods of cooling, temperate Western‐Palearctic species will have likely been restricted to southern refugia in Europe (Iberia, Apennines, and Balkans; Hewitt 1999; but see Thorup et al. 2021). Restrictions to different glacial refugia and subsequent northward expansions during interglacial periods have been linked to contemporary population structure and subspecies divergence in multiple species, including birds and aerial insects (e.g., Schmitt 2007; Hansson et al. 2008; Nadachowska‐Brzyska et al. 2016; de Greef et al. 2022). Occupation of separate glacial refugia by different populations is thought to have driven spatial patterns of genetic differentiation in temperate species, with many Palearctic birds exhibiting contemporary East–West patterns in genetic structure and speciation (e.g., Hansson et al. 2008; Lombardo et al. 2022; Väli et al. 2022).

The European nightjar (*C. europaeus*), henceforth nightjar, is a long‐distance migratory bird with a temperate breeding distribution ranging from Northwest Europe through to East Asia (BirdLife International 2022). Nightjar likely originated from the Afrotropics (Han, Robbins, and Braun 2010), with the most closely related extant species being an Afrotropic resident (Rufous‐cheeked nightjar *Caprimulgus rufigena*) (Han, Robbins, and Braun 2010). Nightjar are composed of six subspecies (*C. europaeus*, *meridionalis*, *sarudnyi*, *unwini*, *plumipes*, *dementievi*) broadly following an East–West clinal distribution (Cleere 1998; Cleere and Christie 2021), although mtDNA analysis has found little association between genetic variation and current subspecies classifications (Schweizer et al. 2020). Migratory behaviour in nightjar was thought to have evolved at the end of the Last Glacial Maximum (LGM; 22 Kya; Larsen et al. 2007), although this remains debated. If nightjars exhibited an Afro‐European migration strategy pre‐LGP, paleoclimatic‐driven periods of dramatic *N*_e_ change should be evident from PSMC analysis, following population expansion and contraction from refugia during global warming and cooling, respectively.

Nightjar have been subject to population decline across the NW of their range (Conway et al. 2007; Langston et al. 2007). Although current population trends are not a cause for concern (IUCN: Least Concern; BirdLife International 2022), nightjar migratory behaviour and habitat specialisation leave them sensitive to environmental change as seen in other taxa (Case, Lawler, and Tomasevic 2015; Bairlein 2016). Its ancestral demographic history may leave nightjar vulnerable to contemporary and future environmental change, if, for example, populations have been subject to bottlenecks resulting in genetic variation depletion (Bürger and Lynch 1995; Frankham, Ballou, and Briscoe 2010; Nadachowska‐Brzyska et al. 2015; Hohenlohe, Funk, and Rajora 2021). The reference genome for the European nightjar was sequenced and assembled in 2021 from a bird captured in Southern Europe during the spring migration period (Secomandi et al. 2021). Here we use this published genome alongside a novel Pacbio HIFI sequence, sequenced by us, sampled from a population from the extreme north‐western range limit in the United Kingdom. We apply PSMC analysis to determine the ancestral demography of nightjar in Europe to estimate the historic *N*_e_ change over time from 10 Kya to 5 Mya. Specifically, we aimed to; firstly, investigate historic *N*_e_ trends of European nightjar populations relative to past climate fluctuations. Secondly, to compare *N*_e_ trends derived from the two genomes to determine whether there was evidence and timing of divergence within the European population. Finally, we utilised temporal *N*_e_ patterns to investigate the timing of the evolution of migratory behaviour in nightjar.

## 2 Methods

### 2.1 Sampling Genetic Material, Extraction and Sequencing

**FIGURE 1:**
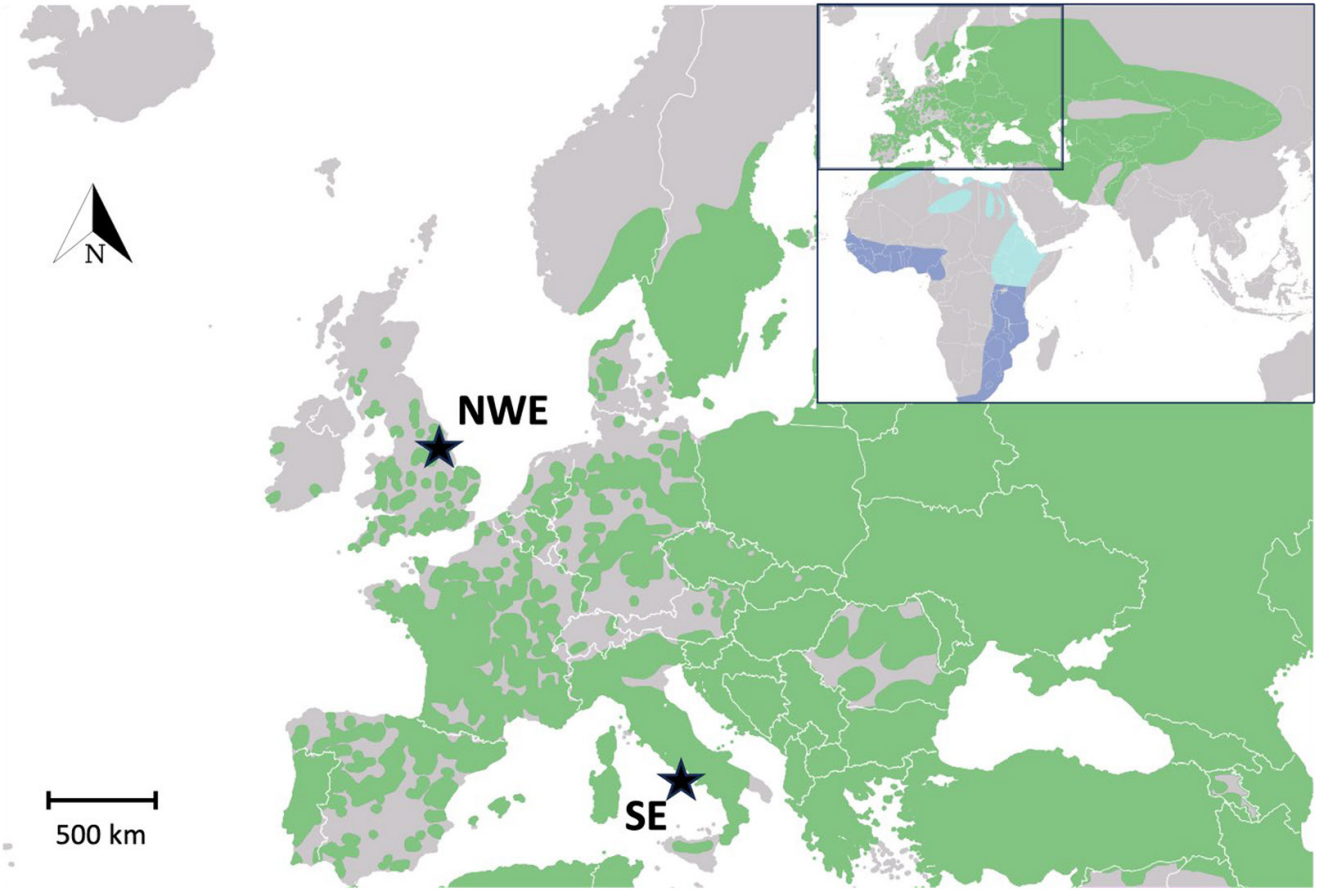
Sampling locations (stars) for European nightjar genomes used in this study, with European breeding distribution (green) overlayed. Species breeding (green), wintering (dark blue) range shown in insert, with locations of migratory stop over shown as light blue. NWE = Northwest European individual; SE = Southern European individual. Original map sourced from IUCN (2023).

A female nightjar from a breeding population in the East of England (latitude: 53.531, longitude: −0.953; Figure 1) was used to extract DNA for sequencing (population henceforth referred to as NW Europe or NWE). The bird died on 7 August 2019, so was assumed to have been part of the breeding population and not moving through on migration (Cramp and Simmons 1985). High molecular weight DNA was extracted from a blood clot in the individual's heart using a modified version of the phenol‐chloroform protocol outlined by Sambrook, Fritsch, and Maniatis (1989). Full extraction protocol details can be found in Appendix S1 and DNA yield and summary statistics in Table S1. The high molecular weight DNA was then sent to the Centre for Genomics Research facility at the University of Liverpool for PacBio HiFi sequencing library preparation. The reference genome (for assembly details see Secomandi et al. 2021) and 10× Genomics Illumina sequence reads were sequenced from a single female nightjar captured in south‐west Italy in spring 2021 (Figure 1) provided by Secomandi et al. (2021) (population henceforth referred to as South Europe or SE).

### 2.2 Genome Alignment

Minimap (minimap2 v. 2.18‐r101; Li, 2018) and BWA mem (arXiv:1303.3997v1 [q‐bio.GN]; Li, 2013) software were used to align clean reads from the NWE (HiFi reads) and SE (10× Illumina reads) nightjars to the reference genome, respectively. Multiply mapped reads were removed and uniquely mapped reads were retained using SAMtools (Li et al. 2009; http://www.htslib.org/). Unmapped reads were then filtered from both files leaving only mapped reads.

### 2.3 PSMC Analysis

To understand ancestral changes in *N*_e_ a partial sequential Markovian coalescent (PSMC) method was applied to the mapped bam files from the HIFI and 10× Illumina reads, for which the average coverage was 30.5× and 88.1×, respectively. First, consensus sequences were generated from the aligned indexed bam files from the HiFi and 10× reads using SAMtools mpileup command and vcfutils.pl. as per Li and Durbin (2011) (https://github.com/lh3/psmc). Consensus files were generated for each chromosome independently before being combined. For the HiFi data, from the NWE genome, four chromosomes (chromosome numbers 3, 5, 25 and 32) failed to produce consensus files and reduced representations for two of the four chromosomes (3 and 5) were used, with two chromosomes (25 and 32) excluded from the analysis. This resulted in a loss of only ~1% of genomic material for analysis. Sex chromosomes were also excluded from the analysis for both Hifi and 10× genomes. This resulted in 89.8% of the NWE genome and 90.8% of the SE genome being retained for downstream analysis. Consensus files were then filtered for read depth and quality. In order to reduce the effects of low coverage and collapsed regions, consensus files were filtered by excluding reads ~< ⅓ and > 2× mean depth using BCFtools (Li 2011; http://www.htslib.org/). This resulted in removing reads < 10× and > 60× for the Hifi data and < 30 and > 120× for the 10× reads, respectively. Finally, filtering for sequence base quality scores of < 20 for the HiFi reads and 10× reads were applied.

The PSMC analysis was then run on the combined consensus .fastq files using the PSMC software package (Li and Durbin 2011; https://github.com/lh3/psmc). PSMC parameters used by Nadachowska‐Brzyska et al. (2015) for demographic analysis of 38 different bird species were chosen for our analysis, where ‘*N*’ (30) is the number of iterations, ‘*t*’ (5) is the maximum time to the most recent common ancestor, ‘*r*’ (5) is the initial mutation/recombination rate (*r* = θ/ρ) and ‘*p*’ (4 + 30 × 2 + 4 + 6 + 10) denotes the distribution of atomic time intervals. In order to determine variation in PSMC predictions, the data were bootstrapped 100 times.

PSMC analysis can be applied to pseudo‐diploid genomes formed from the fusing of haploid genomes from two separate populations or species. When PSMC is applied, deviations in *N*_e_ trends of the pseudo‐diploid genome from the two parent populations can denote reductions in gene flow and points of divergence between the two populations signified by the *N*_e_ of the pseudo‐diploid genome tending towards infinity (reducing coalescence events leading to an apparent increase in *N*_e_) (Li and Durbin 2011; Prado‐Martinez et al. 2013; Sato et al. 2020). To establish whether divergence may have occurred within the European population a pseudo‐diploid genome of both sampled genomes was created. This was achieved by first generating pseudo‐haploid genomes through randomly sampling heterozygous alleles using Seqtk V1.3 ‘randbase’ (*r*) (https://github.com/lh3/seqtk) from both consensus sequence files as generated above. Pseudo‐haploid files were then merged using Seqtk ‘mergefa’ to produce a single pseudo‐diploid genome consensus file. PSMC analysis was then applied to the pseudo‐diploid genome as described above.

Finally, all PSMC results were plotted using gnuplot (http://www.gnuplot.info/) with the R flag applied to export .txt files. In order to plot the PSMC results, the data must be scaled to real‐time by using mutation rate and generation time (Li and Durbin 2011). A generation time of 2 (*g* 2) was selected for nightjars as per Nadachowska‐Brzyska et al. (2015) by multiplying the age of sexual maturity (1) (Cramp and Simmons 1985) by a factor of two (Brommer et al. 2004), with this generation time also used for Chuck‐will's‐widow (*Antrostomus carolinensis*) (Nadachowska‐Brzyska et al. 2015). As no species‐specific mutation rates were available for European nightjar, a mutation rate of *μ* = 4.6 × 10^−9^ nucleotide substitutions per generation was used as per Sato et al. (2020). The mutation rate was initially estimated for collared flycatchers (*Ficedula albicollis*) (Smeds, Qvarnström, and Ellegren 2016), but has since been successfully applied to other passerines (Ericson, Irestedt, and Qu 2022; Gabrielli et al. 2023), raptors (Hanna et al. 2017; Sato et al. 2020) and waterfowl species (Ericson et al. 2017).

## 3 Results and Discussion

**FIGURE 2:**
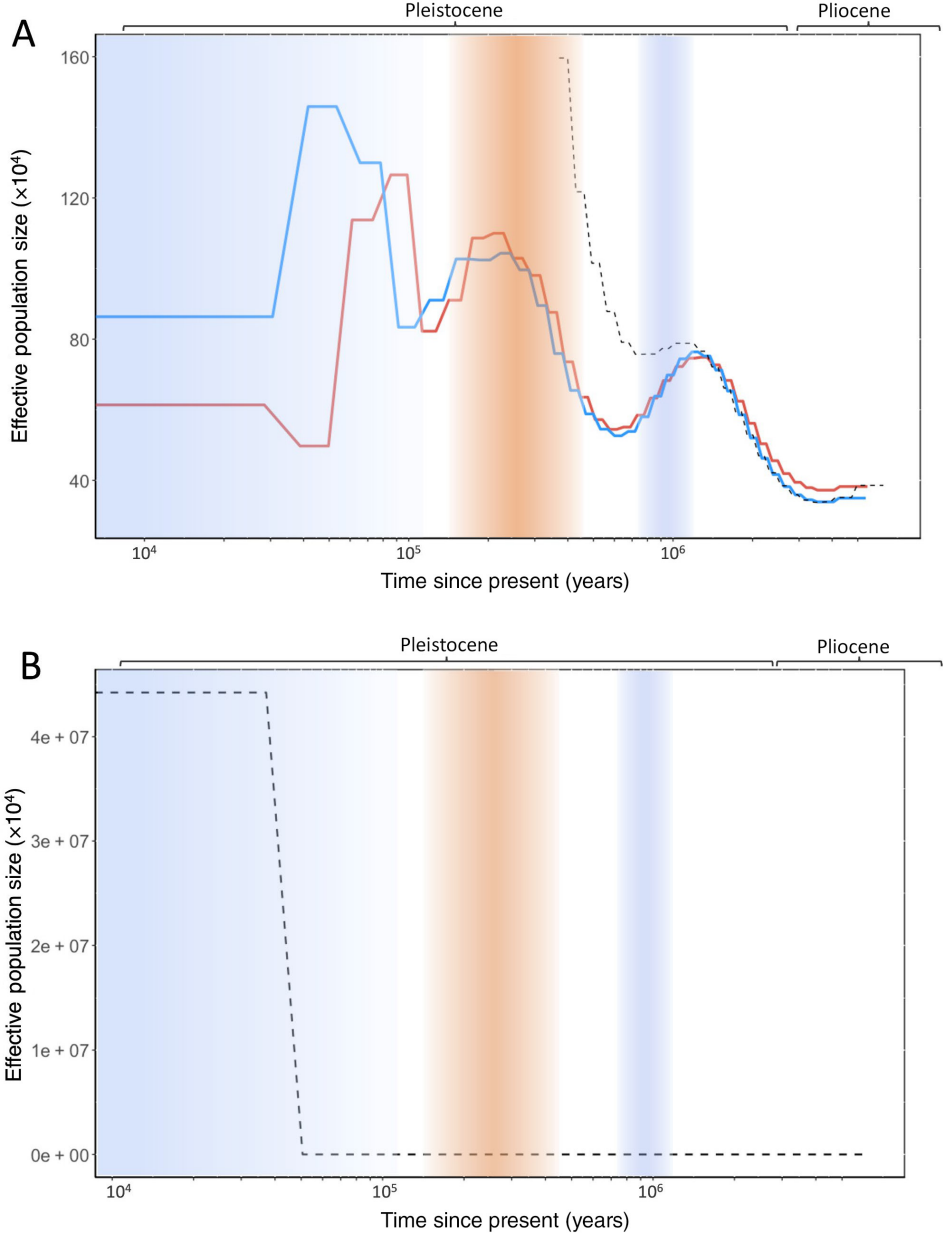
PSMC plots: (A) Effective population sizes (*N*_e_) of NW Europe (red line) and S Europe (blue line) sampled European nightjar, as well as pseudo‐diploid genome of NW/S Europe birds (dashed line), depicting demographic history (*N*_e_ change) over the last ~5 million years (bp), scaled with a mutation rate of 4.6 × 10^−9^ per site and generation time of 2 years. The *x*‐axis depicts time (in years) on a log scale, with the *y*‐axis showing *N*_e_. (B) Estimated *N*_e_ for pseudo‐diploid genome only (dashed line). Approximate timings of significant periods of global climate change are shown by shading along the *x*‐axis. Light blue shading = last glacial period (LGP), orange shading = Mid‐Brunhes Event (MBE) and dark blue shading = Mid‐Pleistocene revolution (MPR).

Using PSMC we found significant fluctuation in *N*_e_ in European nightjar over the last 5 million years, coinciding with major paleoclimatic events (Figure 2A). We found evidence of divergence in the European population ~1.2 Mya (Figure 2A), with final cessation of gene flow found to coincide with the LGP (~110 Kya) (Figure 2B).

### 3.1 Demographic History of European Nightjar

Our analysis suggests that nightjar have experienced significant fluctuations in *N*_e_ over the last ~5 million years. Two of the most significant *N*_e_ changes occurred during the Pleistocene, with *N*_e_ found to increase throughout the early Pleistocene to a maximum of ~780,000 individuals, before decreasing to ~570,000 individuals by 600 Kya during the MPR (~1 Mya–450 Kya; Figure 2A). The *N*_e_ derived from both genomes then increased throughout the MBE to ~1 million individuals by ~240 Kya (Figure 2A) and decreased until ~100 Kya (Figure 2A). At the onset of the LGP *N*_e_ peaked, followed by a steep decline as the LGP progressed (Figure 2A). The NWE and SE effective population sizes then diverged (Figure 2A).

Overall, historic nightjar *N*_e_ in Europe decreased and increased during periods of cooling and warming, respectively (Figure 2A). Nightjar are insectivorous habitat specialists requiring clear fell, heathland, or woodland edge to breed (Cleere 1998), feeding primarily on Lepidoptera (Mitchell et al. 2022). With reductions in temperature and glacial expansion, prey and habitat availability will have been constrained to more southerly latitudes (Schmitt 2007), likely corresponding with a reduction in nightjar distribution and thus *N*_e_. For example, the decrease in nightjar *N*_e_ ~1.2 Mya to ~600 Kya (Figure 2A) overlapped the MPR (~1 Mya–450 Kya), which was characterised by shortened interglacial periods and cooler average temperatures which restricted the northward resurgence of temperate animal and plant communities (Pisias and Moore 1981; Head and Gibbard 2015). Conversely, warmer temperatures will have likely increased the availability of suitable habitat across northerly latitudes (Schmitt 2007; Candy et al. 2010). Indeed, the stable climate of the late Pliocene and early Pleistocene (Head and Gibbard 2015), as well as the short glacial and warm interglacial periods of the MBE (Candy et al. 2010; Barth et al. 2018) associated with increases in nightjar *N*_e_ in our study (Figure 2A). Following similar trends exhibited by other Afro‐Palearctic migrants (e.g., *Ficedcula* flycatchers; Nadachowska‐Brzyska et al. 2016), *N*_e_ of both ancestral nightjar populations greatly decreased as the LGP continued, likely restricting nightjar to Southern European refugia (Schmitt 2007; Lombardo et al. 2022) or North Africa (Thorup et al. 2021). Bootstrapping indicates caution is required regarding exact timings of *N*_e_ fluctuations (Figure S2). However, PSMC analysis in other Caprimulgids (i.e., Chuck‐will's‐widow) and Afro‐Palearctic migrants (i.e., Common cuckoo, *Cuculus canorus*; Nadachowska‐Brzyska et al. 2015), have shown similar fluctuating trends in *N*_e_ over the same timeframe, suggesting that the estimated timings of *N*_e_ change with paleoclimatic events in our study are reasonable.

### 3.2 Divergence in the European Nightjar Population

When applied to a pseudo‐diploid genome (a combination of two individual genomes), PSMC analysis can be used to determine the timing of divergence between ancestral populations. This is signalled by the pseudo‐diploid *N*_e_ trend diverging from that of the two parent genomes and tending towards infinity (Prado‐Martinez et al. 2013). This occurs because coalescence events between the two ancestral populations were severely reduced or ceased, leading to an increase in *N*_e_ as interpreted by the analysis. In our analysis, the pseudo‐diploid *N*_e_ trend appeared to diverge from that of the NWE and SE individuals ~1.2 Mya (Figure 2A). However, true divergence (the point at which *N*_e_ tends to infinity) did not occur until ~40 Kya (Figure 2B). Even taking into account the ~35 Ky error window suggested by the bootstrapping (Figure S2), the main divergence event occurred within the LGP (Figure 2B).

The two nightjar genomes used in this study were sampled from spatially distant sites (Figure 1). However, while the NWE bird can be attributed to a breeding population, the SE bird cannot, having been trapped and sampled during the migration period in SW Italy (Secomandi et al. 2021). Nightjars breeding in Western Europe typically migrate through Iberia during spring migration, with Eastern breeders migrating through Italy and SE Europe, overlapping with the capture location of the SE bird (Evens et al. 2017; Norevik, Åkesson, and Hedenström 2017). If the SE bird's breeding grounds were located in Central or Eastern Europe, as suggested by recent tracking studies (Evens et al. 2017; Norevik, Åkesson, and Hedenström 2017), our results may reflect East–West structure in the European population as in other migratory Palearctic birds (e.g., Lesser whitethroat *Sylvia curruca*; Olsson et al. 2013; Pied wagtail *Motacilla alba*; Li et al. 2016). Indeed, the timing of divergence suggested by our analysis (initial divergence ~1.2 Mya, cessation of gene flow ~40 Kya ± 35 Ky; Figure 2) is consistent with isolation of ancestral populations to glacial refugia during periods of glaciation, a mechanism thought to drive East–West structuring in other Palearctic and Nearctic animal populations periods (Hewitt 2004; Nadachowska‐Brzyska et al. 2016; Yao et al. 2022; de Greef et al. 2022). However, without knowing the breeding location of the SE bird we are unable to make confident inferences on the extent of structure in the European nightjar population from our analysis. Nevertheless, our results do suggest that divergence has occurred within the European nightjar population, with the timings suggesting that periods of glaciation have likely driven this. Further analysis, including PSMC or MSMC applied to one or multiple individuals from known breeding populations across the species European range are needed to better resolve the extent and drivers of structure in the European population.

### 3.3 Evolution of Migratory Behaviour in Nightjar

Following the timeline proposed by our study (Figure 2), nightjar migratory behaviour likely pre‐dated the LGP. The dramatically fluctuating *N*_e_ prior to the LGP throughout the Pleistocene may reflect periods of significant population expansion and contraction associated with climate driven changes in temperate breeding habitat availability (Ponti et al. 2020). Indeed, divergence ~1.2 Mya (Figure 2A) suggested by our analysis is indicative of migratory behaviour persisting throughout the Pleistocene in the species, with Schweizer et al. (2020) also proposing a range wide divergence prior to the LGP (2.4 Mya) for nightjar. Our results tentatively contribute to the growing consensus that long distance migratory behaviour in contemporary Western‐Palearctic avifauna predates the LGP (i.e., Ponti et al. 2020; Ralston et al. 2021; Thorup et al. 2021; Kimmitt et al. 2023).

### 3.4 Limitations of PSMC Analysis

The interpretation of PSMC analysis are influenced by the scaling applied to plots, determined by mutation rate and generation time (Li and Durbin 2011; Mather, Traves, and Ho 2019). However, the effect of these scaling parameters on plots are predictable, shifting the position of the plot along either axis (Nadachowska‐Brzyska et al. 2016). Nevertheless, the pattern of *N*_e_ remains unchanged and independent of the scaling applied (Mather, Traves, and Ho 2019). Data on both mutation rate and generation time are often limited for birds (e.g., see Sato et al. 2020; Chattopadhyay et al. 2019; Ericson, Irestedt, and Qu 2022), including nightjar. Although both parameters are species specific, use of mutation rates from other well‐studied species may be appropriate, with genomic features typically well conserved in birds (Gabrielli et al. 2023). In our study we use the mutation rate of Collared flycatcher (Smeds, Qvarnström, and Ellegren 2016) and a generation time calculated as per Brommer et al. (2004) and Nadachowska‐Brzyska et al. (2015) to scale our PSMC plots. While the mutation rate is for a different species, the parameter has been used successfully for multiple species distantly related from Collared flycatcher (e.g., Passerines; Ericson, Irestedt, and Qu 2022; Gabrielli et al. 2023; raptors; Hanna et al. 2017; Sato et al. 2020; waterfowl; Ericson et al. 2017). Caution must then be applied concerning the exact timings and magnitudes of *N*_e_ change presented in this study, as highlighted by bootstrapping (see Figures S1 and S2). In the future, when nightjar‐specific mutation rates are available, re‐application and scaling of PSMC may produce more accurate timings and magnitude of *N*_e_ change and provide valuable support for the interpretation of our results. However, the overall pattern of *N*_e_ will likely remain unchanged (Li and Durbin 2011; Nadachowska‐Brzyska et al. 2016; Mather, Traves, and Ho 2019).

Lastly, the small sample size used for PSMC analysis (single diploid genome) can incur limitations, specifically leading to inaccurate *N*_e_ trends over recent time scales (1–20 Kya; Li and Durbin 2011; Mather, Traves, and Ho 2019) owing to the low number of recombination events over very recent time scales (Sheehan, Harris, and Song 2013). Nevertheless, single genomes have been readily used to infer the demographic history of entire species (Nadachowska‐Brzyska et al. 2015, 2016; Brüniche‐Olsen et al. 2021; Termignoni‐Garcia et al. 2022), subspecies (Sato et al. 2020; Tsujimoto et al. 2023; Dalapicolla et al. 2024) and populations (Preckler‐Quisquater et al. 2023; Lichman et al. 2024). Where populations have experienced different demographic histories, these will be highlighted by PSMC from a single member of those populations (Li and Durbin 2011). Indeed, in cases where multiple individuals from the same population have been sampled, the demographic trends have remained concordant among those individuals (e.g., Nadachowska‐Brzyska et al. 2016; Sato et al. 2020; Preckler‐Quisquater et al. 2023). Therefore, despite the small sample size used in this study we are confident that the differing *N*_e_ trends between the two nightjars are correct, with this inference bolstered by the results from our pseudo‐diploid analysis. Moreover, the sampling strategy employed here reflects the availability of sequenced nightjar genomes (*n* = 2 at the time of writing), with our work providing an initial insight into the demographic history in the species. Future research should look to extend the analysis to individuals sampled from across the entirety of nightjars breeding range. This would enable an initial investigation into genetic structure and underlying ancient demography and help place our results in context. Furthermore, increasing the sample size within each sampling locality may help to bolster confidence of population‐specific findings from PSMC, although it is unlikely that differences in *N*_e_ trends will be found among individuals from the same ancestral population.

## 4 Conclusion

PSMC is a useful tool to characterise past demography and timing of differentiation within species or populations, processes which underlie contemporary genetic and demographic patterns. Results from our PSMC analysis suggest that nightjar were highly susceptible to climatic variation, increasing in number during warm interglacials and long periods of relative climate stability. Our analysis suggests that divergence has occurred within the European nightjar population over the last ~1 million years, with geneflow cessation after ~40 Kya. Our results also suggest that migratory behaviour in nightjar evolved prior to the LGP, persisting throughout the Pleistocene. The historical context provided by our research suggests that the current climate is likely suitable for nightjar. Habitat loss, fragmentation, degradation and disturbance are reported as the primary drivers of contemporary population reduction in nightjar (Langston et al. 2007; Lowe, Rogers, and Durrant 2014; Ashpole et al. 2015). Although nightjar have been shown to persist through historic climate change, contemporary anthropogenic pressures may reduce the ability of the species to adapt to the current rapidly changing climate.

## Author Contributions

**George Day:** conceptualization (lead), formal analysis (lead), funding acquisition (lead), investigation (lead), methodology (lead), software (lead), visualization (lead), writing – original draft (lead). **Graeme Fox:** formal analysis (supporting), methodology (supporting), software (equal). **Helen Hipperson:** data curation (supporting), methodology (supporting), resources (supporting), software (equal). **Kathryn H. Maher:** formal analysis (supporting), methodology (supporting), software (equal). **Rachel Tucker:** methodology (supporting), resources (equal). **Gavin J. Horsburgh:** methodology (supporting), resources (equal). **Dean Waters:** conceptualization (equal), supervision (supporting), writing – review and editing (supporting). **Kate L. Durant:** supervision (supporting), writing – review and editing (supporting). **Kathryn E. Arnold:** conceptualization (equal), funding acquisition (equal), project administration (supporting), supervision (lead), validation (supporting), writing – original draft (supporting), writing – review and editing (lead). **Terry Burke:** conceptualization (equal), funding acquisition (equal), investigation (equal), project administration (equal), resources (equal), supervision (equal), writing – review and editing (supporting). **Jon Slate:** conceptualization (equal), methodology (supporting), software (supporting), supervision (equal), visualization (supporting), writing – original draft (supporting), writing – review and editing (supporting).

## Conflicts of Interest

The authors declare no conflicts of interest.

## Supporting information


Appendix S1.

## Data Availability

The sequence reads from the UK European nightjar genome sequenced for this study are available from the GenBank database under BioProject: PRJNA1162521.
