# Supplementary material for: Revealing the Demographic History of the European Nightjar (Caprimulgus europaeus)
Source: Ecol Evol. 2024 Oct 26;14(10):e70460. doi: 10.1002/ece3.70460 (PMC11512156; doi:10.1002/ece3.70460)
Supplement: Supplementary file 1 — Appendix S1. [file ECE3-14-e70460-s001.docx]

Revealing the Demographic History of the European nightjar (*Caprimulgus europaeus*).

Supporting Information

Sample extraction

The 40μl blood sample was initially stored in 500 μl SET buffer and frozen. Pre-extraction the sample was then thawed at 37° before being spun down, before adding 10 μl RNase (100 mg/ml), and after resting the sample for 2 minutes at room temperature (RT). After which, 13 μl of SDS (20%) was added to the sample and then let to rest 30 min at 37° for continued RNase treatment. 7.5μl of proteinase K (~20mg/ml) was then added and mixed well before being spun down. The sample was then transferred to a water bath (55°C) and left overnight, mixing the sample after one hour. The sample was then spun down again using a centrifuge before adding 50μl of 5M NaCl Spin down, which was mixed and again spun down using a centrifuge. 540μl of phenol was added to the sample mixing well until the solution forms a homogenous mix. The sample was then left to rest for

40-60 minutes at RT under a fume hood and mixed every 5 minutes. Samples were then transferred to a centrifuge and spun down at 10,000 rpm for 15 minutes. 500μl of chloroform/isoamyl alcohol (24:1) was added to a new set of 3mL glass tubes. The supernatant from the phenol samples was then removed and transferred to the tubes containing the chloroform, this was mixed well and centrifuged at 10,000 rpm for 15 minutes. 50μl of NaAc (3M) was added to a new 1.5mL tube before transferring the supernatant from the chloroform stage tubes to the new tubes containing the NaAc. 100μl of 95% ethanol chilled on ice was then added to the sample before mixing well. Mixing was continued until precipitation of DNA was observed. A pipette was used to remove the precipitated DNA from the sample. The precipitated DNA was then rinsed in ice-cold 70% ethanol three times. The DNA was then air dried for 3-minutes until all of the ethanol had evaporated. The DNA was then dissolved in 100μl of 0.1x TE (pH7.8), the sample was kept at 4°C overnight prior to quantification using a Qubit fluorometer (Invitrogen), subsequent dilution and size determination using 0.8% agarose gel at low voltage (max 50V for 1.5h) against lambda DNA (see Table S1). The sample was then sorted at -80°C until PacBio library preparation was required.

Sample Yields and Summary Statistics:

Table

S1 summary statistics from sample extraction and HiFi sequencing.

| ID | Yield (ng/μl) | Mean HiFi read length (bp) | Number of HiFi Reads | Number of HiFi Bases | Hifi Median Accuracy | Median Number of HiFi passes distribution |
| --- | --- | --- | --- | --- | --- | --- |
| *NWE* | 96 | 14918 | 1386719 | 35605959186 | Q31 | 9 |

Bootstrapped PSMC plots


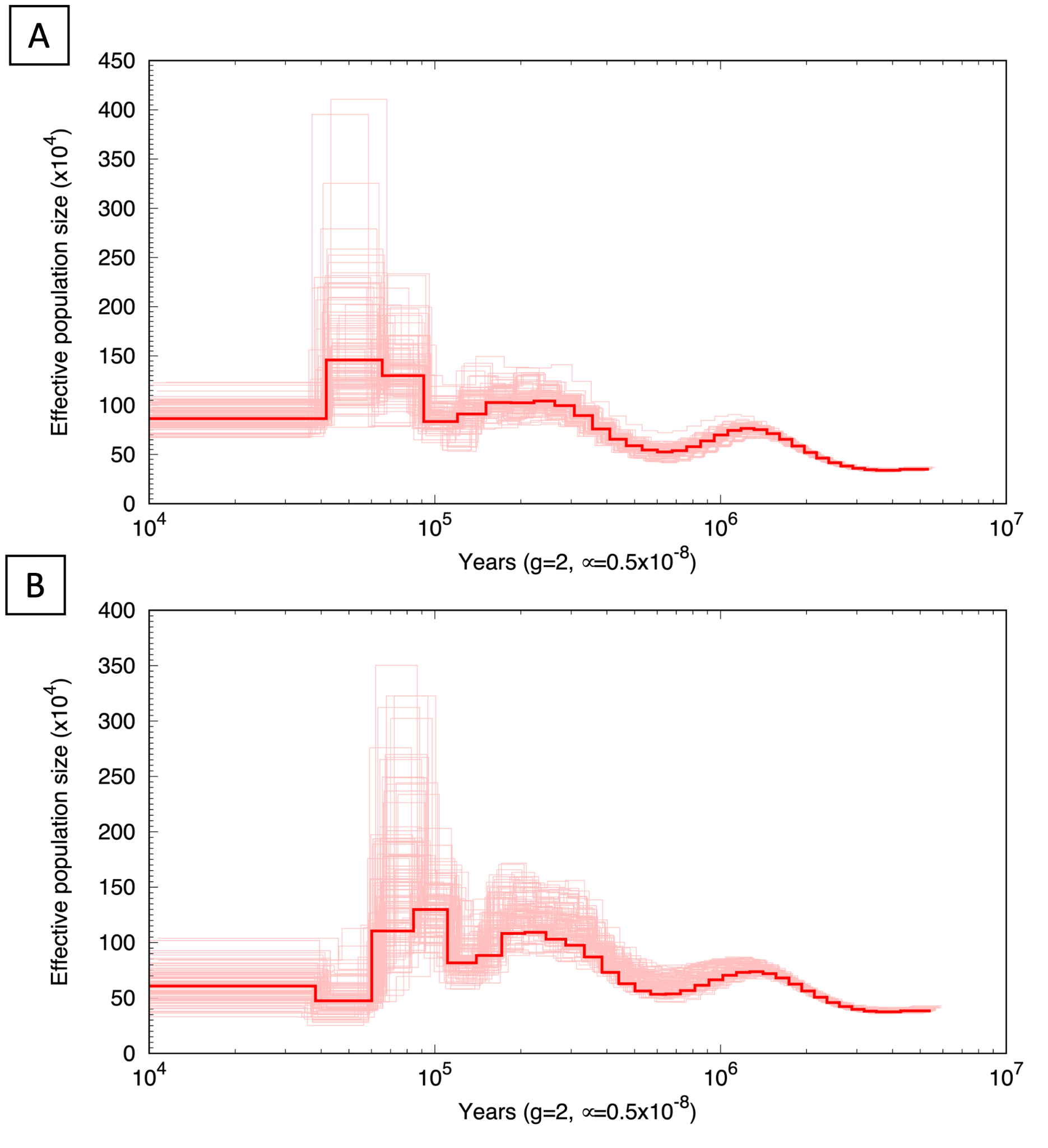


Fig S1, Bootstrapped PSMC plot for SE (A) and NWE (B) sampled European nightjar depicting demographic historic (Ne change) over the last ~5 million years (bp), scaled with a mutation rate of 4.6 × 10−9 per site and generation time of 2 years. The X-axis depicts time (in years) on a log scale, with the Y-axis showing effective population size. The red line shows effective population size estimate, with the light pink lines depicting PSMC estimates for 100 bootstrapped sequences.


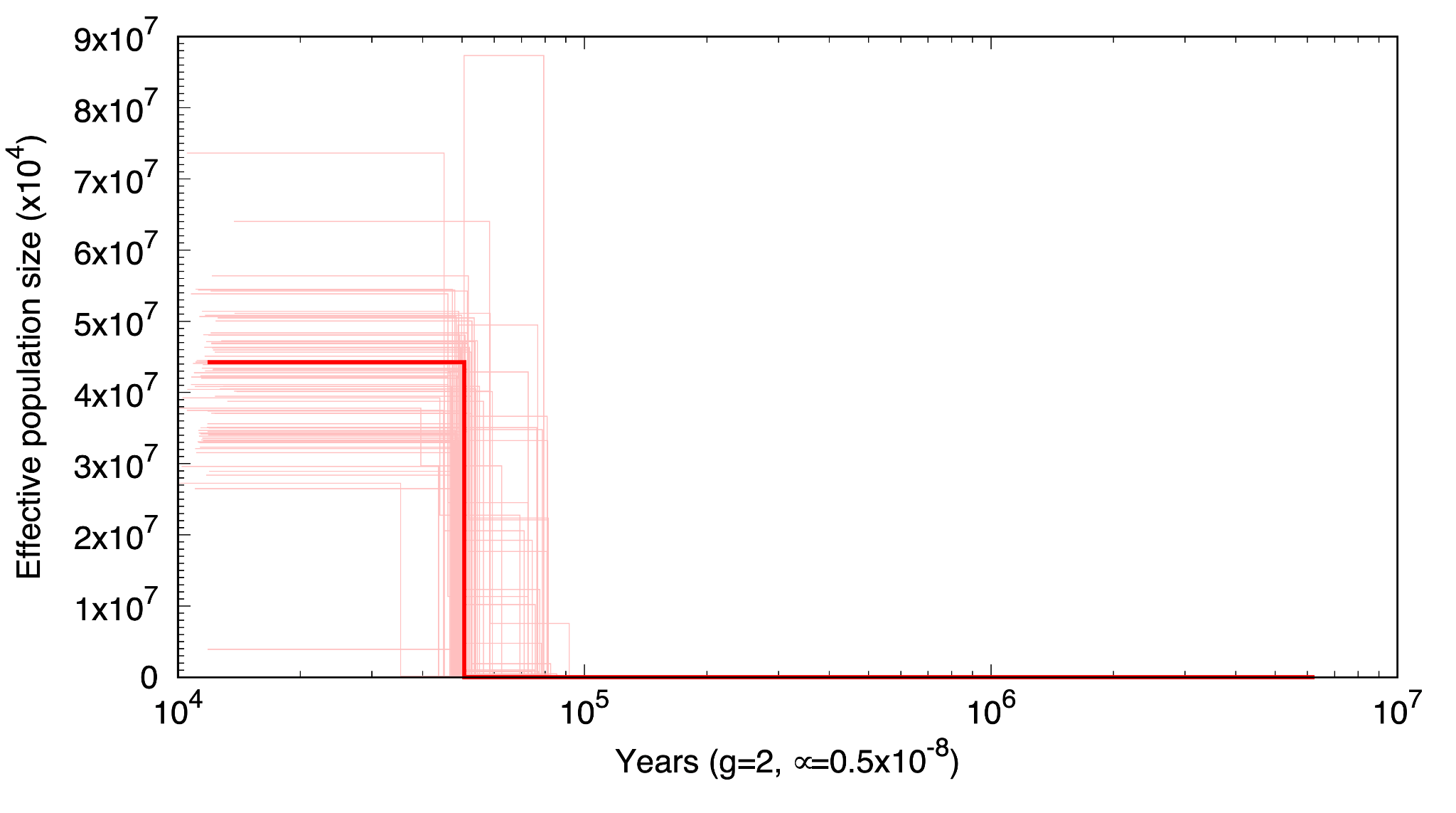


Fig S2, Bootstrapped PSMC plot for pseudo-diploid genome of the NW and S European sampled populations. The X-axis depicts time (in years) on a log scale, with the Y-axis showing effective population size. The red line shows the effective population size estimate, with the light pink lines depicting PSMC estimates for 100 bootstrapped sequences.
